# Can exercise regulate the relationship between noise pollution and the perception of physical and mental health among Chinese adults? An empirical study based on CGSS 2021

**DOI:** 10.3389/fpubh.2025.1594917

**Published:** 2025-08-14

**Authors:** Peng Shi, Xiaosu Feng, Shanshan Lyu

**Affiliations:** ^1^School of Physical Education, Shandong University of Technology, Zibo, China; ^2^School of Physical Education, Liaoning Normal University, Dalian, China; ^3^School of Physical Education, China University of Mining and Technology, Xuzhou, China; ^4^Faculty of Liberal Arts, Humanities and Culture, Perdana University, Serdang, Malaysia

**Keywords:** noise pollution, exercise, physical and mental health, China, moderating effect

## Abstract

**Objective:**

To explore the relationship between noise pollution, exercise, and the perception of physical and mental health among Chinese adults, to test the moderating effect of exercise on the relationship between noise pollution and physical and mental health perception, and to provide a basis for the formulation of environmental health policies and public health policies.

**Methods:**

Using 2,717 data points from the 2021 China General Social Survey (CGSS), data analysis was conducted using SPSS 25.0, Stata 12.0 software, and GraphPad Prism 8 software. The Mann–Whitney *U* test, Kruskal-Wallis test, contingency table *χ*^2^ test, multiple linear regression analysis, binary Logistic regression analysis, and Generalized Linear Model (GLM) were employed.

**Results:**

The average age of the subjects was 52.04 ± 17.64 years, including 54.8% women. After controlling for related confounding factors, the high noise pollution perception group had lower perception of physical health (PPH; *β* = −0.135, 95%*CI* = -0.231 ~ −0.039, *p* < 0.01) and perception of mental health (PMH; *β* = −0.151, 95%*CI* = −0.240 ~ −0.062, *p* < 0.01). The regular exercise group had higher PPH (*β* = 0.224, 95%*CI* = 0.146 ~ 0.342, *p* < 0.01), PMH (*β* = 0.093, 95%*CI* = 0.001 ~ 0.184, *p* < 0.01), and perception of physical and mental health (PPMH; *β* = 0.236, 95%*CI* = 0.137 ~ 0.334, *p* < 0.01). Exercise has a significant moderating effect on the relationship between noise pollution and physical and mental health perception (*p* < 0.05), with regular exercisers generally having higher PPH, PMH, and PPMH.

**Conclusion:**

The high noise pollution perception group has lower physical and mental health perception; the regular exercise group has higher physical and mental health perception. Regular exercise can counteract the lower physical and mental health perception caused by higher noise pollution.

## 1 Introduction

Noise pollution is one of the main sources of pollution affecting human physical and mental health, and is a common challenge faced by humanity. It has become an increasingly severe global environmental health issue and an important global public health problem (1). Especially with the rapid advancement of industrialization and urbanization, traffic noise, industrial noise, and construction noise in urban environments continue to expand (2), constantly interfering with the quality of life of local residents and causing a series of health problems. A report released by the European Environment Agency (3) points out that noise pollution has become an important environmental health issue in Europe, where road noise is the main source, and more than 125 million Europeans are troubled by road noise above 55 decibels. Noise pollution not only causes specific damage to the auditory system but also affects people’s emotions, sleep, and work efficiency (4, 5). In addition, relevant statistical data (3) shows that noise pollution causes about 8 million people to have sleep disorders, leads to about 43,000 people being hospitalized for treatment each year, and is associated with more than 10,000 premature deaths. Therefore, noise pollution not only has a negative impact on the physical and mental health of the nation but also creates an economic burden for the development of the country and society.

Although noise pollution and its hazards have received widespread attention and research in developed countries, they have not been given sufficient importance in developing countries, including China (6). The reason may be that developing countries are more focused on economic development, which may come at the expense of the environment. Moreover, policymakers may be more concerned with seemingly more urgent issues such as poverty, unemployment, and infrastructure construction, and have not yet had the energy to focus on the control of noise pollution sources. China is the largest developing country in the world, and due to rapid industrialization over the past few decades, people have been increasingly exposed to noise pollution in their daily work environments, transportation, and leisure activities. For example, the noise levels in labor-intensive industries such as construction and manufacturing are much higher than those in office environments; the use of motor vehicles has made the streets noisy and bustling; and leisure activities such as listening to rock music and singing karaoke are filled with a lot of noise. Xie et al. (7) pointed out that noise pollution is the fourth major inducement of sub-health status in China’s population, in addition to long-term mental tension, fatigue, frequent overtime work, and air pollution. In addition, noise pollution has also been proven to be the third major determinant of hypertension prevalence in China, after family history and salt intake (8). The potential health threats of noise pollution are gradually drawing the attention of researchers and policymakers, especially as the Chinese government’s understanding of sustainable development goals increases, it is particularly important to explore effective intervention measures or mitigation strategies.

Exercise is a widely recommended positive lifestyle that has been extensively proven to play an important role in improving physical and mental health (9). For example, Wong et al. (9) found that exercise can improve the physical function of Chinese older adults, improve their balance and body composition, reduce the risk of falling, reduce negative emotions such as depression and anxiety, and improve quality of life and happiness. Jansen (10) found that noise exposure can induce peripheral vasoconstriction in exercising individuals, which may reduce muscle blood supply and affect exercise performance. However, based on the positive benefits of exercise, it may moderate the association between noise pollution and physical and mental health, that is, regular exercise may reduce the negative effects of noise pollution. In addition, from the perspective of environmental psychology, chronic noise pollution can induce the hypothalamic–pituitary–adrenal (HPA) axis to remain in a continuously activated state, thus leading to continuously high levels of stress hormones such as cortisol (11). The long-term high levels of stress hormones like cortisol have a negative impact on the body’s immune system (12), can interfere with metabolism, resulting in abnormal blood pressure and blood sugar levels (13, 14), and can also cause emotional problems such as anxiety and depression (15). However, relevant studies (16, 17) has found that moderate exercise can regulate the function of the HPA axis, reduce the activity of the HPA axis, and regulate the secretion of stress hormones such as cortisol, thereby reducing the adverse effects of chronic noise pollution on the body. But currently, there is a lack of research on exercise and noise pollution, and some researchers have also called for the exploration of the comprehensive effects of noise pollution and lifestyle factors such as exercise on human health.

Developing countries, including China, do not have sufficient indoor exercise venues, and most residents exercise outdoors. Outdoor exercise can enhance physical and mental health, but it may also increase the risk of noise exposure, which puts people in a dilemma (18). Therefore, it is particularly important to study the comprehensive effect of exercise and noise pollution on health, or the moderating effect of exercise on the association between noise pollution and health. Based on this, this study uses the Chinese General Social Survey (CGSS) database in 2021 to explore the relationship between noise pollution and the perception of physical and mental health among Chinese adult groups, and to analyze the potential role of exercise in alleviating the physical and mental problems induced by noise pollution. Through this study, it is hoped to draw the attention of all sectors of society to the problem of noise pollution and promote the development of environmental health promotion. In addition, the results of this study will also provide a scientific basis for the formulation of public health policies, and provide references for how to improve the quality of life of urban residents and promote the construction of Healthy China.

## 2 Data sources and methods

### 2.1 Data sources

The CGSS is the earliest national, comprehensive, and continuous academic survey project in China. The database systematically and comprehensively collects data at multiple levels, including society, community, family, and individual, providing support for summarizing the trends of social change and exploring topics of significant scientific and practical importance (19). The CGSS employs a rigorous multi-stage stratified random sampling method to select samples from 31 provincial administrative units in mainland China. During the process, contact with participants is established through telephone communication, followed by on-site visits and information collection (20). The sampling hierarchy includes three levels, with residential areas or counties as the first-level sampling units, villages or urban neighborhood communities as the second-level sampling units, and households as the third-level sampling units, with one person selected from each household (21). Sampling is stratified according to socioeconomic and demographic indicators, with sampling probabilities proportional to size (21). The CGSS 2021 conducted a comprehensive and detailed survey of the perceived level of noise pollution among Chinese residents for the first time, providing solid data support for the conduct of this study. The CGSS 2021 collected a total of 2,717 data points on Chinese residents’ perceptions of noise pollution and physical and mental health, which serves as the basis for this study. Additionally, after excluding invalid responses from participants (such as “do not know,” “not applicable,” etc.) and missing data, this study used linear interpolation to fill in the missing values. Currently, CGSS data has become the most important data source for studying Chinese society and is widely used in scientific research, teaching, and government decision-making. Many studies have used CGSS data to explore China’s environmental pollution issues (22) and residents’ physical and mental health issues (23), which have been proven to be detailed and reliable.

### 2.2 Variables and instruments

#### 2.2.1 Independent variable

The independent variable in this study is noise pollution. CGSS 2021 used a four-level self-assessment method to investigate the severity of noise pollution at the locations where the participants lived. Here, 1 represents “very serious,” 2 represents “relatively serious,” 3 represents “not very serious,” and 4 represents “not serious at all.” For the purpose of facilitating subsequent statistical analysis, this study re-coded these values: the original 1 was re-coded as 4 (very severe); the original 2 was re-coded as 3 (severe); the original 3 was re-coded as 2 (not severe); and the original 4 was re-coded as 1 (not severe at all). Additionally, the re-coded values were combined into two categories: values 1 and 2 were merged into “low pollution,” and values 3 and 4 were merged into “high pollution.” The subjective perception of being insensitive to noise can reflect the impact of noise actually experienced and felt by individuals in their daily lives, and can eliminate the interference of noise sensitivity. In addition, the investigation of the subjectively perceived noise pollution is relatively simple and easy to conduct, and the cost is also low. A large amount of data can be quickly collected in large-scale surveys. Promoting the convergence between the public’s subjective perception and the objective environment is conducive to the public making objective expectations and psychological anticipations regarding the future environment, and realizing the maximization of environmental pollution prevention and control policies. Compared with the perception of the objective environment, subjective environmental perception plays a more important role in predicting the residents’ life satisfaction, physical and mental health (24). Numerous existing studies (25, 26) have proven that the respondents’ perception level of environmental pollution is highly correlated with the objectively measured environmental pollution directly, that is, the subjective perception of the environment is consistent with the objectively detected environmental index in terms of valence. In addition, the current evaluation method of such subjective perception has been widely applied in the research on promoting environmental health (27, 28).

#### 2.2.2 Dependent variables

The dependent variables in this study include perception of physical health (PPH), perception of mental health (PMH), and perception of physical and mental health (PPMH).

1. PPH. The CGSS 2021 used a five-level self-rating method to ask respondents about their inability to complete expected work or daily activities due to health problems in the past 4 weeks. The scale ranges from 1 to 5, indicating always to never, respectively. Thus, higher scores indicate better physical health. PPH is a subjective assessment of one’s own health status and is one of the globally recognized indicators of health level (29, 30). It is convenient to obtain and has high validity in China (31).
2. PMH. The CGSS 2021 asked respondents to answer questions about their mental health status, such as depression, anxiety, and frustration in the past 4 weeks, based on the following two descriptive items: ① “Because of emotional problems, you were unable to complete expected work or daily activities”; ②“Because of emotional problems, you were distracted from your work or daily activities.” The CGSS 2021 used a five-level self-rating method, where 1 to 5 indicates always to never, respectively. Higher scores indicate better mental health. In this study, principal component analysis (PCA) was used to extract one principal component, with a cumulative variance contribution rate of 85.98%, which is sufficient to describe the mental health status of the population (32).
3. PPMH. The CGSS 2021 used a five-level self-rating method to ask respondents about the impact of physical or emotional problems on social activities (such as visiting friends or relatives) in the past 4 weeks. The scale ranges from 1 to 5, indicating always to never, respectively. Thus, higher scores indicate better physical and mental health.

#### 2.2.3 Moderator variable

The moderator variable in this study is physical exercise. The CGSS 2021 used a five-point scale to ask respondents how often they engaged in physical exercise during their leisure time in the past year, where 1 indicates “every day”; 2 indicates “several times a week”; 3 indicates “several times a month”; 4 indicates “several times a year or less”; and 5 indicates “never.” Firstly, drawing on previous studies (33, 34), this study divided the frequency of physical exercise into two categories: no exercise and exercise. Specifically, a score of 5 was classified as no exercise, while scores of 1 to 4 were classified as exercise. Secondly, referring to the concept of the sports-engaged population in China (35), which defines individuals who exercise at least three times a week with each session lasting more than 30 min at a moderate intensity, this study further divided exercise into irregular exercise and regular exercise. Scores of 3 and 4 were classified as irregular exercise, while scores of 1 and 2 were classified as regular exercise. This type of self - reported exercise survey has been widely used in numerous studies (36, 37) and is extensively applied in Chinese databases such as the CGSS.

#### 2.2.4 Control variables

This study refers to previous studies (33, 38) and selects the following control variables: age, gender, region of residence, place of residence, educational level, body mass index (BMI), and socio-economic status (SES). (1) Age. The CGSS 2021 inquired about the respondents’ dates of birth. This study calculates the age of the respondents by subtracting their birth year from 2021. (2) Gender. This study assigns a value of 1 to “male” and a value of 2 to “female.” (3) Region of residence. The CGSS 2021 surveyed the provinces where the respondents reside. Referring to the geographical regional division standards of China (39), this study categorizes the provinces into three regions: eastern, central, and western. The eastern region includes 12 provinces, municipalities, and autonomous regions: Beijing, Tianjin, Hebei, Liaoning, Shanghai, Jiangsu, Zhejiang, Fujian, Shandong, Guangdong, Guangxi, and Hainan. The central region includes 9 provinces and autonomous regions: Shanxi, Inner Mongolia, Jilin, Heilongjiang, Anhui, Jiangxi, Henan, Hubei, and Hunan. The western region includes 10 provinces, municipalities, and autonomous regions: Chongqing, Sichuan, Guizhou, Yunnan, Tibet, Shaanxi, Gansu, Qinghai, Ningxia, and Xinjiang. These regions are assigned values of 1, 2, and 3, respectively. (4) Place of residence. This study assigns a value of 1 to “urban” and a value of 2 to “rural.” (5) Educational level. Referring to previous study (40), this study assigns a value of 1 to “no formal education, private school, literacy class, elementary school,” which is categorized as “primary school or below”; a value of 2 to “junior school”; a value of 3 to “vocational school, regular school, secondary vocational school, technical school,” which is categorized as “high school, secondary vocational school, technical school”; and a value of 4 to “associate degree, bachelor’s degree, graduate degree or above,” which is categorized as “college or above.” (6) BMI. The CGSS 2021 surveyed the height and weight of the respondents. This study calculates the BMI using the formula BMI = weight (kg) / height (m)^2^. (7) SES. The CGSS 2021 used a ten-point scale to survey the social stratum in which the respondents currently reside. Higher scores indicate higher SES.

### 2.3 Mathematical statistics

In this study, data processing and statistical analysis were conducted using SPSS 25.0 and Stata 12.0 software, while GraphPad Prism 8 software was employed for the visualization of statistical results. Continuous variables were described using mean (*M*) and standard deviation (*SD*), and categorical variables were described using frequency and percentage (%). Firstly, the normality of the perceived mental and physical health data was tested using the one-sample Kolmogorov–Smirnov test combined with P–P and Q-Q plots. The results indicated that the data did not follow a normal distribution (*p* < 0.01). Therefore, the Mann–Whitney *U* test and Kruskal-Wallis test were used for intergroup comparison analysis of PPH, PMH, and PPMH data. Secondly, since the data does not follow a normal distribution, this study uses the Generalized Linear Model (GLM) for analysis. After controlling for confounding factors, the linear response model within the GLM is employed to conduct a multiple linear regression analysis, exploring the relationships between noise pollution and PPH, PMH, and PPMH, respectively. Thirdly, the relationship between exercise and PPH, PMH, and PPMH, respectively, after controlling for confounding factors, was examined using the linear response model in the GLM. In the study, the Omnibus test was used to evaluate the overall goodness of fit of the models, and Wald’s *χ*^2^ test was employed for model effect testing. Additionally, pairwise comparison analysis was used to estimate the marginal means of multi-category independent variables. Finally, an interaction term between noise pollution and exercise was constructed, and the linear response model in GLM was used to explore the relationship between exercise and PPH, PMH, and PPMH after controlling for relevant variables, and to test the potential moderating effect of exercise on the relationship between noise pollution and the dependent variables. In the visualization of the results, noise pollution was used as the between-group variable, and physical exercise as the within-group variable for plotting. The significance level for all statistical tests involved in this study was defined as *α* = 0.05.

## 3 Results

### 3.1 Basic information about the participants

The 2,717 participants had a mean age of (52.04 ± 17.64) years. They were mainly from the eastern (45.4%), central (34.6%), and western (20.0%) regions of China. The participants included 55.9% urban residents and 44.1% rural residents, as well as 45.2% males and 54.8% females. Among the participants included, 33.4% have an educational attainment of primary school or below; 28.3% have a junior school education; 18.0% have a high school education; and 20.3% have a college education or above. Among the participants, 35.4% did not exercise, 24.1% exercised irregularly, and 40.4% exercised regularly. The proportion of participants who perceived low noise pollution was 77.6%, while those who perceived high noise pollution accounted for 22.4%. In addition, the average BMI of the participants was (23.07 ± 3.74) kg/m^2^, which was within the normal range as a whole. The average SES of the participants was roughly at a slightly lower than middle level (4.28 ± 1.81), and the average scores of PPH, PMH and PPMH were generally at a slightly higher than middle level. Detailed demographic information of the participants is shown in Table 1.

**Table 1:** Basic information about the participants.

Continuous variables	*M*	*SD*
Age	52.04	17.64
BMI (kg/m^2^)	23.07	3.74
SES	4.28	1.81
PPH	3.91	1.17
PMH^PCA^	0.00	1.00
PPMH	3.97	1.07

Categorical variables	Frequency	Percentage (%)
Exercise		
No exercise	963	35.4
Irregular exercise	655	24.1
Regular exercise	1,099	40.4
Noise pollution		
Low	2,109	77.6
High	608	22.4
Gender		
Male	1,228	45.2
Female	1,489	54.8
Region of residence		
Eastern	1,234	45.4
Central	940	34.6
Western	543	20.0
Place of residence		
Urban	1,518	55.9
Rural	1,199	44.1
Educational level		
Primary school or below	907	33.4
Junior school	770	28.3
High school	489	18.0
College or above	551	20.3

### 3.2 Intergroup comparative analysis of perception of physical and mental health

Firstly, the PMH of the low-pollution perception group was significantly higher than that of the high-pollution perception group (*Z* = −2.138, *p* = 0.033). However, there was no statistically significant difference between the two groups in terms of PPH (*Z* = −0.317, *p* = 0.751) and PPMH (*Z* = −0.685, *p* = 0.493). Secondly, the PMH (*Z* = −4.578, *p* < 0.001) and PPMH (*Z* = −5.146, *p* < 0.001) of the regular exercise group were significantly higher than those of the irregular exercise group. The PPH (*Z* = −10.346, *p* < 0.001), PMH (*Z* = −5.769, *p* < 0.001), and PPMH (*Z* = −6.617, *p* < 0.001) of the regular exercise group were also significantly higher than those of the non-exercise group. The PPH of the irregular exercise group was significantly higher than that of the non-exercise group (*Z* = −7.631, *p* < 0.001). The results of the intergroup comparison analysis of PPH, PMH, and PPMH are detailed in Table 2.

**Table 2:** Intergroup comparative analysis of PPH, PMH, and PPMH.

Variables	Low-pollution		High-pollution		*Z*	*P*
	*M* ± *SD*	Rank mean	*M* ± *SD*	Rank mean		
PPH	3.90 ± 1.18	1356.56	3.93 ± 1.15	1367.46	−0.317	0.751
PMH	0.02 ± 1.00	1375.83	−0.07 ± 1.01	1300.60	−2.138	0.033
PPMH	3.98 ± 1.08	1364.25	3.95 ± 1.07	1340.80	−0.685	0.493

Variables	No exercise (*M* ± *SD*)	Irregular exercise (*M* ± *SD*)	Regular exercise (*M* ± *SD*)	*Z*	*P*
PPH	3.55 ± 1.31#&	4.09 ± 0.97#	4.12 ± 1.07&	117.205	<0.001
PMH	−0.14 ± 1.11&	−0.02 ± 0.84*	0.13 ± 0.98&*	38.977	<0.001
PPMH	3.79 ± 1.21&	3.95 ± 0.92*	4.14 ± 1.00&*	50.657	<0.001

# indicates a significant difference between the no exercise and irregular exercise groups; & indicates a significant difference between the no exercise and regular exercise groups; * indicates a significant difference between the irregular exercise and regular exercise groups.

### 3.3 Association between noise pollution and perception of physical and mental health

After controlling for relevant confounding factors, compared with the low-pollution perception group, the high-pollution perception group had lower PPH (*β* = −0.135, 95% *CI* = −0.231 to-0.039, *p* < 0.01) and PMH (*β* = −0.151, 95% *CI* = −0.240 to-0.062, *p* < 0.01). However, the relationship between the two groups was not significant for PPMH (*β* = −0.084, 95% *CI* = −0.181 to 0.012, *p* > 0.05). Among the control variables, age was significantly negatively correlated with PPH and PPMH (*p* < 0.01); compared with males, females had lower PPH and PMH (*p* < 0.01); compared with residents in the eastern region, those in the western and central regions had lower PPH, PMH, and PPMH (*p* < 0.01); compared with urban residents, rural residents had lower PPH and PMH (*p* < 0.01); compared with residents with primary school education or below, those with junior high school education had higher PPH, PMH, and PPMH (*p* < 0.01), and those with senior high school education or above had higher PPH and PPMH (*p* < 0.01); SES was significantly positively correlated with PPH, PMH, and PPMH (*p* < 0.01); BMI was not significantly correlated with PPH, PMH, and PPMH (*p* > 0.05). The results of the GLM linear response model for the associations between noise pollution and PPH, PMH, and PPMH among Chinese residents are shown in Table 3.

**Table 3:** Results of the GLM linear response model for the associations between noise pollution and PPH, PMH, and PPMH.

Variables	PPH		PMH		PPMH	
	*β*	95*CI*	*β*	95%*CI*	*β*	95%*CI*
Noise pollution						
High	−0.135**	(−0.231, −0.039)	−0.151**	(−0.240, −0.062)	−0.084	(−0.181, 0.012)
Age	−0.022**	(−0.025, −0.019)	−0.002	(−0.004, 0.000)	−0.001**	(−0.011, −0.006)
Gender						
Female	−0.158**	(−0.239, −0.077)	−0.171**	(−0.246, −0.096)	−0.055	(−0.136, 0.026)
Region of residence						
Central	−0.113*	(−0.204, −0.021)	−0.194**	(−0.278, −0.109)	−0.122**	(−0.213, −0.031)
Western	−0.171**	(−0.281, −0.061)	−0.230**	(−0.332, −0.128)	−0.177**	(−0.288, −0.066)
Place of residence						
Rural	−0.242**	(−0.332, −0.153)	−0.131**	(−0.214, −0.048)	−0.085	(−0.175, 0.005)
Educational level						
Junior school	0.205**	(0.099, 0.313)	0.164**	(0.066, 0.264)	0.141**	(0.034, 0.248)
High school	0.219**	(0.088, 0.349)	0.201**	(0.080, 0.322)	0.053	(−0.078, 0.184)
College or above	0.226**	(0.080,0.373)	0.181**	(0.046, 0.317)	0.024	(−0.121, 0.172)
BMI	0.009	(−0.002, 0.019)	0.005	(−0.005, 0.015)	0.010	(−0.000, 0.021)
SES	0.081**	(0.058, 0.103)	0.052**	(0.031, 0.072)	0.050**	(0.027, 0.072)

*p < 0.05; **p < 0.01.

### 3.4 Association of exercise and perception of physical and mental health

After controlling for relevant variables, compared with the non-exercise group, the regular exercise group had higher PPH (*β* = 0.224, 95% *CI* = 0.146 to 0.342, *p* < 0.01), PMH (*β* = 0.093, 95% *CI* = 0.001 to 0.184, *p* < 0.01), and PPMH (*β* = 0.236, 95% *CI* = 0.137 to 0.334, *p* < 0.01). Pairwise comparison analysis revealed that the regular exercise group had significantly higher PPH, PMH, and PPMH than the irregular exercise group (*p* < 0.05). However, there were no statistically significant differences in PPH, PMH, and PPMH between the irregular exercise group and the non-exercise group (*p* > 0.05). Among the control variables, age was significantly negatively correlated with PPH and PPMH (*p* < 0.01). Compared with males, females had lower PPH and PMH (*p* < 0.01). Compared with residents in the eastern region, those in the western and central regions had lower PPH, PMH, and PPMH (*p* < 0.01). Compared with urban residents, rural residents had lower PPH and PMH (*p* < 0.01). Compared with residents with primary school education or below, those with junior high school education had higher PPH, PMH, and PPMH (*p* < 0.01), and those with senior high school education or above had higher PPH and PPMH (*p* < 0.01). Compared with junior high school graduates, university graduates or above had lower PPMH (*p* < 0.05). SES was significantly positively correlated with PPH, PMH, and PPMH (*p* < 0.01). BMI was not significantly correlated with PPH, PMH, and PPMH (*p* > 0.05). The results of the GLM linear response model of exercise and PPH, PMH, PPMH among Chinese adult groups are detailed in Table 4.

**Table 4:** Results of the GLM linear response model for the associations between exercise and PPH, PMH, and PPMH.

Variables	PPH		PMH		PPMH	
	*β*	95%*CI*	*β*	95%*CI*	*β*	95%*CI*
Exercise						
Irregular exercise	0.080#	(−0.033, 0.192)	−0.064#	(−0.169, 0.040)	−0.009#	(−0.122, 0.103)
Regular exercise	0.244**#	(0.146, 0.342)	0.093*#	(0.001, 0.184)	0.236**#	(0.137, 0.334)
Age	−0.022**	(−0.025, −0.019)	−0.002	(−0.005, 0.000)	−0.009**	(−0.012, −0.006)
Gender						
Female	−0.150**	(−0.230, −0.069)	−0.168**	(−0.243, −0.093)	−0.048	(−0.129, 0.032)
Region of residence						
Central	−0.107*	(−0.198, −0.016)	−0.195**	(−0.280, −0.111)	−0.120**	(−0.211, -0.029)
Western	−0.166**	(−0.275, −0.056)	−0.231**	(−0.333, −0.130)	−0.174**	(−0.284, −0.065)
Place of residence						
Rural	−0.195**	(−0.284,−0.106)	−0.102*	(−0.184, −0.019)	−0.046	(−0.135, 0.043)
Educational level						
Junior school	0.177**	(0.070, 0.284)	0.156**	(0.056, 0.255)	0.114*&	(0.006, 0.221)
High school	0.166*	(0.034, 0.298)	0.188**	(0.066, 0.311)	0.007	(−0.125, 0.139)
College or above	0.157*	(0.008,0.305)	0.163*	(0.025,0.302)	−0.035&	(−0.184, 0.114)
BMI	0.008	(−0.003, 0.018)	0.005	(−0.005, 0.015)	0.010	(−0.001, 0.020)
SES	0.077**	(0.054, 0.099)	0.051**	(0.031, 0.072)	0.046**	(0.024, 0.068)

*P < 0.05; **P < 0.01; # indicates that the difference in PPH, PMH, or PPMH between the regular exercise group and the irregular exercise group is statistically significant; & indicates that the difference in PPH, PMH, or PPMH between residents with university education.

### 3.5 Test of the moderating role of exercise in the association between noise pollution and perception of physical and mental health

**Figure 1:**
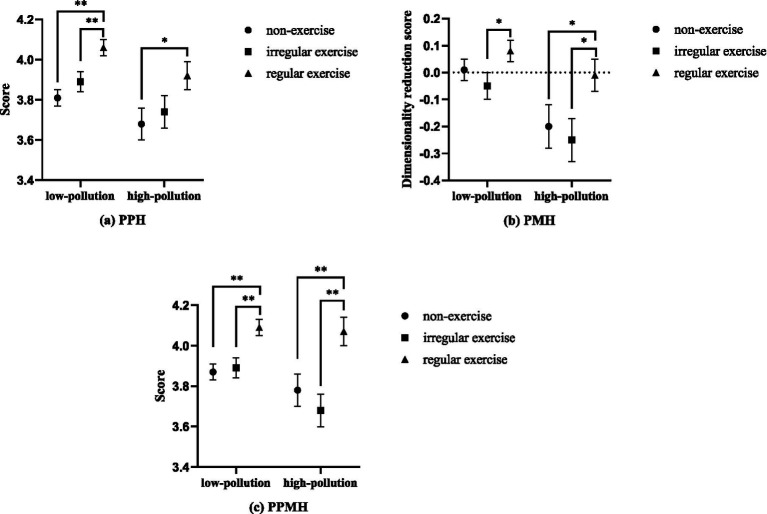
Results of the moderation analysis of exercise in the relationship between noise pollution and perceived mental and physical health. ^*^P < 0.05; ^**^P < 0.01.

Firstly, regarding PPH, the interaction effect between noise pollution and exercise was significant (*χ*^2^ = 34.264, *df* = 5, *p* < 0.001). Specifically, among the low-pollution perception group, the regular exercise group had higher PPH (4.06 ± 0.04) compared with both the non-exercise (3.81 ± 0.04) and irregular exercise (3.89 ± 0.05) groups (*p* < 0.01); among the high-pollution perception group, the regular exercise (3.92 ± 0.07) group had higher PPH compared with the non-exercise group (3.68 ± 0.08; *p* < 0.05). Secondly, regarding PMH, the interaction effect between noise pollution and exercise was also significant (*χ*^2^ = 24.668, *df* = 5, *p* < 0.001). Specifically, among the low-pollution perception group, the regular exercise (0.08 ± 0.04) group had higher PMH compared with the irregular exercise group (−0.05 ± 0.05; *p* < 0.05); among the high-pollution perception group, the regular exercise (−0.01 ± 0.06) group had higher PMH compared with both the non-exercise (−0.20 ± 0.08) and irregular exercise (−0.25 ± 0.08) groups (*p* < 0.05). Lastly, regarding PPMH, the interaction effect between noise pollution and exercise was significant (*χ*^2^ = 38.070, *df* = 5, *p* < 0.001). Specifically, among the low-pollution perception group, the regular exercise (4.09 ± 0.04) group had higher PMH compared with both the non-exercise (3.87 ± 0.04) and irregular exercise (3.89 ± 0.05) groups (*p* < 0.05); among the high-pollution perception group, the regular exercise (4.07 ± 0.07) group had higher PMH compared with both the non-exercise (3.78 ± 0.08) and irregular exercise (3.68 ± 0.08) groups (*p* < 0.05). In summary, exercise plays a significant moderating role in the relationship between noise pollution and perceived mental and physical health. Regardless of whether individuals perceive low or high pollution, those who engage in regular exercise consistently have higher PPH, PMH, and PPMH. In other words, regular exercise can mitigate the negative impact of higher noise pollution on perceived mental and physical health. The results of the moderation analysis of exercise in the relationship between noise pollution and perceived mental and physical health are detailed in Figure 1.

## 4 Discussion

### 4.1 Highly polluted perceived groups have lower PPH and PMH

The results of this study found that individuals with a high perception of pollution had lower PPH and PMH, which is similar to previous studies (6, 41–43). The potential physiological explanations for this correlation mainly include the following six pathways. Firstly, noise pollution is a stressor. Continuous exposure to noise can induce an increase in the concentration of stress hormones such as catecholamine, cortisol, and adrenaline (44, 45), leading to enhanced cardiovascular activity, elevated blood pressure, myocardial ischemia, and myocardial damage (46), which in turn give rise to a series of physical and mental health problems. Secondly, chronic exposure to noise can trigger a chronic inflammatory response, leading to the activation of vascular endothelial cells and inflammation (47, 48). This inflammatory state is associated with an increased risk of various diseases, including hypertension, heart disease, and stroke. At the molecular level, noise pollution can reduce the activity of natural killer (NK) cells, promote lymphocyte proliferation, and induce changes in the expression levels of pro-inflammatory cytokines such as TNF-*α* and IL-1β (49, 50), thereby contributing to the development and progression of diseases. Thirdly, noise pollution may lead to impaired physiological functions of vascular endothelial cells, resulting in a decreased capacity to maintain vascular tension, promote blood cell circulation, regulate platelet activity, and modulate inflammatory responses. This can lead to endothelial dysfunction (51), which in turn may increase the risk of cardiovascular diseases such as heart disease. Fourthly, noise pollution may increase oxidative stress in the vascular system and the brain (52), leading to the accumulation of excess free radicals. This, in turn, can directly damage myocardial cells and vascular endothelial cells, resulting in myocardial cell dysfunction and endothelial dysfunction, thereby contributing to the occurrence and progression of heart disease (53). Fifthly, exposure to noise can activate the sympathetic nervous system and reduce the activity of the parasympathetic nervous system, leading to sustained increases in heart rate and blood pressure (54). This increased cardiac burden can have adverse effects on overall health. Finally, long-term exposure to noise, especially nighttime noise, can lead to reduced sleep duration and decreased sleep quality, and may even cause sleep disorders (55). Sleep is an important period for the body’s recovery, and chronic sleep disorders can lead to a variety of health problems (56).

However, this study has not yet identified a potential link between noise pollution and PPMH. The value of PPMH is not obtained by simply adding the values of PPH and PMH, but is derived from separate items of inquiry. Although PPMH is related to PPH and PMH to some extent, the interplay of physical and psychological factors may affect researchers’ overall assessment of respondents’ health. For example, happiness and life satisfaction may not be entirely correlated with PPH, as individuals with higher levels of happiness and life satisfaction may report higher PPMH. Therefore, this holistic concept of health assessment is not consistent with the research findings of PPH and PMH. Moreover, given the limitations of the measurement tools, this study may not be able to fully capture the complexity of respondents’ health conditions. Thus, further exploration is needed to clarify the relationship between the two.

### 4.2 Regular exercise groups have higher PPH, PMH and PPMH

The results of this study found that individuals who exercise regularly have higher levels of PPH, PMH, and PPMH, which is similar to previous studies (36, 57, 58). These studies have all shown a significant positive correlation between physical exercise and the physical and mental health of respondents. However, the relationship between the two may not be a simple linear one, but rather a nonlinear relationship. For example, Xu et al. (59) found that the frequency, duration, and energy expenditure of physical exercise have a U-shaped relationship with mental health.

In addition, relevant stage-based models in health psychology, such as the Trans theoretical Model, the Health Behavior Process Model, and the Berlin Exercise Stage Model, suggest that the behavior change of engaging in physical exercise is a process that is stage-based, nonlinear, dynamic, and complex. It is recommended that research be conducted by categorizing individuals based on the characteristics of different exercise groups to explore the health promotion benefits of behavior and the factors influencing behavioral change (60). Based on this, relevant studies (61, 62) have divided respondents into two categories—“exercisers” and “non-exercisers”—and found that compared with the non-exercising group, the exercising group has higher levels of PPH, PMH, and subjective well-being. However, according to the perspective of the stage-based models (63), there are significant differences in exercise intentions and behaviors within the exercising group, with some individuals having high exercise intentions and engaging in regular exercise, while others have low exercise intentions and do not exercise regularly. Therefore, the differences between these two exercise groups may have confounded the research results. In light of this, this study adopted the perspective of the stage-based models and divided the exercise groups into three categories: “regular exercisers,” “irregular exercisers,” and “non-exercisers.” The study found positive benefits associated with regular exercise. Additionally, it was found that the irregular exercise group may not have shown any positive benefits in physical and mental health due to the lack of continuous and sufficient physical stimulation. Therefore, to gain more positive benefits from exercise, it is recommended to develop the habit of regular exercise.

### 4.3 Regular exercise can reverse the lower perceived physical and mental health associated with higher noise pollution

The results of this study show that, regardless of whether it is the high pollution perception groups or the low pollution perception groups, individuals who exercise regularly always have higher levels of PPH, PMH, and PPMH. This exploratory finding reveals that regular exercise can counteract the lower perceived physical and mental health caused by higher noise pollution.

The positive benefits of physical exercise on perceived physical and mental health have been widely confirmed and are elaborated in the above discussion. According to research on air pollution, although air pollution has adverse effects on physical exercise and health (21, 64), maintaining moderate exercise can still offset potential health problems caused by air pollution to some extent, especially in terms of cardiopulmonary function (65–67). The negative health impacts of noise pollution share certain similarities with those of air pollution. For instance, both can reduce sleep quality (68), as well as diminish overall physical and mental health and life satisfaction (69, 70). Therefore, given these commonalities, exercise can offset potential health problems caused by noise pollution to some extent. Li (68) revealed the possible mechanism by which exercise counteracts noise and promotes physical and mental health from an immunological perspective. This study (68) explored the effects of moderate-intensity exercise on the concentrations of inflammatory cytokines such as IL-6, IL-8, and TNF in noise-stressed rats, finding that moderate-intensity exercise has significant protective benefits on the immune function of rats under both normal and noise-stress conditions. Moreover, green exercise provides another explanation for this result. Although exposure to outdoor environments may involve more noise pollution, exposure to green spaces is more likely to evoke positive emotions, enhance the pleasure of exercise, and alleviate daily anxiety and fatigue, thus providing a buffering effect on physical and mental health (71, 72). Huang et al. (73) confirmed this view, finding that green exposure can reduce mental health problems caused by environmental pollution and enhance the mental health benefits of physical exercise.

### 4.4 The value and significance of this study

This study focuses on noise pollution, exercise, and the perceived physical and mental health of Chinese adults, providing valuable and multifaceted evidence and direction for the formulation of public health policies.

Firstly, by highlighting the dangers of noise pollution, it promotes the development of environmental governance policies. The study clearly indicates that a high perception of noise pollution is closely associated with lower perceived physical and mental health among adults. This finding helps policymakers fully recognize the serious threat of noise pollution to public health, thereby encouraging them to develop stricter noise control policies, enhance supervision of noise emissions in industries such as manufacturing, transportation, and construction, and effectively reduce noise pollution to ensure the quality of residents’ living environments. This approach addresses the root causes of noise pollution’s adverse effects on public health.

Secondly, by emphasizing the health-promoting effects of exercise, it supports the improvement of health promotion policies. The study confirms that regular exercise significantly enhances the perceived physical and mental health of adults. This provides strong scientific evidence for public health policies aimed at encouraging and supporting residents’ participation in physical exercise. Policymakers can use this information to develop relevant policies, such as increasing investment in public sports facilities, building more parks, fitness trails, and community gyms, and reducing the costs for residents to engage in exercise. They can also organize diverse sports activities to attract residents of different ages and fitness levels and conduct public education campaigns to raise awareness of the benefits of exercise and enhance residents’ motivation to exercise. This creates a positive atmosphere for physical activity, encourages regular exercise habits among residents, and improves overall health levels.

Lastly, by highlighting the moderating effect of exercise on noise pollution, it supports the development of comprehensive intervention policies. The study also finds that regular exercise can effectively mitigate the negative impact of high noise pollution on perceived physical and mental health. Based on this, public health policies can integrate noise pollution control with exercise promotion to develop comprehensive intervention strategies. For example, in areas with severe noise pollution, soundproofing facilities can be planned and constructed, along with exercise venues that encourage residents to mitigate the harm of noise pollution through physical activity. Additionally, research on exercise methods and precautions in noisy environments can be conducted, and the findings can be incorporated into public health campaigns and guidelines to provide scientific advice for residents on how to maintain their physical and mental health through appropriate exercise in noisy conditions.

### 4.5 Limitations of this study

This study initially reveals the relationship between noise pollution, exercise, and the perceived physical and mental health as well as the prevalence of cardiovascular diseases among Chinese adults, which is instructive for the formulation of public health policies. However, the study still has the following limitations. (1) The CGSS 2021 required participants to subjectively assess noise pollution, physical activity, mental and physical health, and the prevalence of cardiovascular diseases. There may be discrepancies between the accuracy of the study results and objective measurements. Moreover, the use of single-item self-reported measures may involve measurement errors, which could affect the accuracy of the results. Therefore, it is recommended that future studies employ more objective data for analysis to validate the findings of this study. (2) There may be a time-lag effect in the association between noise pollution, exercise, and the prevalence of cardiovascular diseases, which may require long-term longitudinal studies for verification. (3) Due to the limitations of the study design, it is not possible to determine the causal relationships between the above variables. Follow-up studies using longitudinal designs are needed to explore the internal associations between these variables. (4) The findings of this study are primarily applicable to the specific cultural and social context of China, and their generalizability may be significantly limited. Based on this, it is recommended that future studies incorporate datasets from multiple countries with diverse cultural backgrounds to enhance the generalizability of the sample, thereby drawing more universally applicable conclusions.

## 5 Conclusion

This study, based on the 2021 CGSS data, delves into the relationship between noise pollution, exercise, and the perceived physical and mental health of Chinese individuals. The results show that groups with higher perceived noise pollution have lower levels of PPH and PMH, as well as a higher prevalence of cardiovascular diseases. In contrast, groups that engage in regular exercise have higher levels of PPH, PMH, and PPMH. Additionally, this study examines the moderating role of exercise in the relationship between noise pollution and perceived health. The findings indicate that regular exercise can significantly offset the negative impact of high noise pollution on perceived health. This study is one of the first analyses based on large-scale data to reveal that exercise has a significant moderating effect on the negative health impact of noise pollution. This finding not only enriches research in the relevant field but also provides new perspectives and directions for future studies and practices. The results of this study also provide a scientific basis for the formulation of public health policies and offer important guidance for improving the quality of life of residents.

## Data Availability

Publicly available datasets were analyzed in this study. This data can be found here: Data supporting the results of this study are available from CGSS 2021, but these data cover a wide range of Chinese socio-economic data, so many are not applicable to this study. Upon reasonable request and with permission from CGSS 2021, data can be obtained from the respective authors. The original data from CGSS 2021 are detailed at http://www.cnsda.org/index.php?r=projects/view&id=65635422.
